# On the Complementarity of Sparse L0 and CEL0 Regularized Loss Landscapes for DOA Estimation

**DOI:** 10.3390/s21186081

**Published:** 2021-09-10

**Authors:** Alice Delmer, Anne Ferréol, Pascal Larzabal

**Affiliations:** 1 Thales, 92230 Gennevilliers, France; anne.ferreol@satie.ens-cachan.fr; 2 SATIE, ENS Paris-Saclay, Université Paris-Saclay, CNRS, 91190 Gif-sur-Yvette, France; pascal.larzabal@satie.ens-cachan.fr

**Keywords:** direction-of-arrival, sparse modeling, L0 regularization, nonconvex regularization, loss landscapes

## Abstract

L0 sparse methods are not widespread in Direction-Of-Arrival (DOA) estimation yet, despite their potential superiority over classical methods in difficult scenarios. This comes from the difficulties encountered for global optimization on hill-climbing error surfaces. In this paper, we explore the loss landscapes of L0 and Continuous Exact L0 (CEL0) regularized problems in order to design a new optimization scheme. As expected, we observe that the recently introduced CEL0 penalty leads to an error surface with less local minima than the L0 one. This property explains the good behavior of the CEL0-regularized sparse DOA estimation problem for well-separated sources. Unfortunately, CEL0-regularized landscape enlarges L0-basins in the middle of close sources, and CEL0 methods are thus unable to resolve two close sources. Consequently, we propose to alternate between both error surfaces to increase the probability of reaching the global solution. Experiments show that the proposed approach offers better performance than existing ones, and particularly an enhanced resolution limit.

## 1. Introduction

The study of Direction-Of-Arrival (DOA) estimation has a long history in signal processing. Conventional methods [1] such as beamforming or Capon’s method are still the subject of numerous works, e.g., [2]. However, they present degraded performance in the presence of multiple close sources. Subspace-based methods such as MUltiple SIgnal Classification (MUSIC) have been introduced to improve the resolution limit for multiple sources. Unfortunately, these methods fail in presence of correlated sources [3]. They also often require a priori knowledge of the number of sources and need a sufficient number of snapshots. Sparse DOA estimation has received much attention in the last decade due to its potential performance in such scenarios [4,5,6,7,8,9,10].

Sparsity naturally arises in DOA estimation when considering a discretization of the field-of-view in numerous candidate angles of arrival on a grid. The aim is to estimate a vector γ whose dimension covers the whole grid and whose only the very few entries corresponding to sources DOA are non-zero.

The purpose of sparse estimation, under the Single Measurement Vector (SMV) framework, is to retrieve the sparse vector $\gamma \in C^{G}$ from the noisy measurement y=Bγ+w, with $y\in C^{N^{2}}$, $N^{2}\ll G$, knowing a dictionary B. The dictionary B depends on the array’s responses for the different angles of arrival candidates. It can be formulated as the following regularized problem:

(1) $$\min_{\gamma }\left\{J_{\ell_{0}}\left(\lambda ,\gamma \right)=\frac{1}{2}{∥B\gamma -y∥}_{2}^{2}+\lambda {∥\gamma ∥}_{0}\right\},$$

where the so-called $\ell_{0}$-norm is defined as ${∥\gamma ∥}_{0}=\mathrm{Card}\left\{g\in \left\{1,\ldots ,G\right\}:\gamma_{g}\neq 0\right\}$, $\gamma_{g}$ being the the *g*-th component of vector γ. The $\ell_{0}$-norm is the natural measure of sparsity: it counts the number of non zero components of the vector. The regularization parameter λ aims to balance the relative importance between the data fidelity term ${1/2∥B\gamma -y∥}_{2}^{2}$ and the $\ell_{0}$-norm enforcing sparsity of the solution. The sparse estimation problem can also be formulated as a constraint problem. The relationship between the two $\ell_{0}$-problems has been studied in [11]. Based on this study, recent theoretical results [12,13] have been provided for an off-line selection of λ so that the $\ell_{0}$-problems are equivalent. The regularization parameter is here chosen in accordance with those results.

The $\ell_{0}$-minimization problem is known to be NP-hard: its resolution usually requires an exhaustive search. The use of the very recently proposed global optimization method [14] is limited to small size problems and is thus unadapted here because of its huge computational cost. So far, many suboptimal methods have therefore been proposed, as the well-known Iterative Hard Thresholding (IHT) algorithm. IHT is a proximal gradient descent algorithm: it iteratively produces estimates ${\hat{\gamma }}^{\left(i\right)}$ so that the cost function $J_{\ell_{0}}$ decreases, starting from an initial point ${\hat{\gamma }}^{\left(0\right)}$. However, the $\ell_{0}$-regularized error surface $J_{\ell_{0}}$ exhibits numerous local minima, and convergence is only proved to a stationary point. Convex relaxation of (1) by the $\ell_{1}$-norm is also a popular alternative. However, conditions [15] under which the sparse vector can be reliably recovered are usually too restrictive for practical applications as in DOA estimation.

More recently, minimization of a regularized criterion using nonsmooth nonconvex but continuous penalties has drawn considerable attention [16], and it has been shown in many applications that it can yield significantly better performance than with using the $\ell_{1}$-norm [17]. Such penalties include $\ell_{q}$-norms (0<q<1), Smoothly Clipped Absolute Deviation (SCAD), and Minimax Concave Penalty (MCP) [18]. The Continuous Exact $\ell_{0}$ (CEL0) penalty [19] corresponds to the limit case of MCP for the SMV framework. CEL0 is shown to suppress some local minima of $J_{\ell_{0}}$ while preserving the global one. The CEL0-regularized cost function is:

(2) $$J_{\mathrm{CEL}0}\left(\lambda ,\gamma \right)=\frac{1}{2}{∥B\gamma -y∥}_{2}^{2}+\Phi_{\mathrm{CEL}0}\left(\lambda ,\gamma \right),$$

with

(3) $$\begin{array}{cc} \; & \Phi_{\mathrm{CEL}0}\left(\lambda ,\gamma \right)=\sum_{i\in I_{G}}\phi \left(\lambda ,\alpha_{i},\gamma_{i}\right) \end{array}$$

(4) $$\begin{array}{cc} \; & \phi \left(\lambda ,\alpha_{i},\gamma_{i}\right)=\lambda -\frac{{\left(|\gamma_{i}|-\sqrt{2\lambda \alpha_{i}}\right)}^{2}}{2\alpha_{i}}1_{\left\{|\gamma_{i}|\leq \sqrt{2\lambda \alpha_{i}}\right\}} \end{array}$$

and αi=1∥B·,i∥22, $B_{\cdot ,i}$ being the *i*-th column of matrix B. 𝟙 is the indicator function whose value is one if the given condition is respected and zero otherwise. Despite its promising interest, we have shown in [12] that traditional suboptimal optimization schemes of CEL0-regularized functional as Iterative Reweighted $\ell_{1}$ (IRL1) or Forward Backward (FB) are unable to resolve close sources.

The aim of this paper is to investigate the properties of $J_{\ell_{0}}$ and $J_{CEL0}$ loss surfaces in order to propose a sparse optimization strategy to resolve close sources. The goal is to improve the resolution limit of both MUSIC method, limited for low Signal-to-Noise Ratios, and existing sparse methods. The proposed approach follows an iterative scheme that requires little computational cost. It shows good performance for close sources and does not require any particular initialization.

Outline of the paper: we first explain the model and the sparse DOA estimation problem in Section 2. In Section 3, we compare $J_{\ell_{0}}$ and $J_{\mathrm{CEL}0}$ loss surfaces in the context of multi-source DOA estimation: an in-deep analysis of the minimizers is provided for two close sources. Based on this analysis, Section 4 presents the proposed optimization scheme, whose originality is to alternate between both loss surfaces. Numerical simulations of Section 5 finally show the validity and advantages of our approach.

Notations: Upper-case and lower-case boldface letters denote matrices and vectors, respectively. $\cdot^{*}$ denotes the conjugate, $\cdot^{T}$ the transpose and $\cdot^{H}$ the conjugate transpose of a vector or matrix. $x_{i}$ is the $i^{th}$ component of vector x, and $\omega_{i}$ the $i^{th}$ component of the set ω. Given a matrix X, the $i^{th}$ column is denoted $X_{\cdot ,i}$. Considering a matrix X of dimension N×G, $X_{\omega }$ is the submatrix of X containing the columns indicated by the set $\omega \subseteq I_{G}$, where $I_{G}=\left\{1,\ldots ,G\right\}$ is the ordered index set. Similarly, $x_{\omega }$ is the subvector of x defined as: $x_{\omega }={\left(x_{\omega_{1}},\ldots ,x_{\omega_{♯\omega }}\right)}^{T}$, with ♯ω the number of elements in ω.

## 2. Sparse DOA Estimation Problem

### 2.1. On-Grid Array Signal Modeling

Consider *M* far field narrow band sources impinging an array of *N* antennas from angles ${\tilde{\theta }}_{m}$, m=1…M. For a single snapshot at time *t*, the output array signal $x\left(t\right)\in C^{N}$ is expressed as $x\left(t\right)={\left[x_{1}\left(t\right)\ldots x_{N}\left(t\right)\right]}^{T}=\sum_{m=1}^{M}a\left({\tilde{\theta }}_{m}\right){\tilde{s}}_{m}\left(t\right)+n\left(t\right)$, where $a({\tilde{\theta }}_{m})$ is the steering vector (or array response) for the direction ${\tilde{\theta }}_{m}$, ${\tilde{s}}_{m}\left(t\right)$ the complex envelope of the signal of the *m*th source, and $n\left(t\right)\in C^{N}$ a white gaussian noise vector of covariance $E\left[n\left(t\right)n^{H}\left(t\right)\right]=\sigma_{n}^{2}I_{N}$, where $I_{N}$ is the N×N identity matrix. Let us suppose the directions of the sources are part of a predefined set $\Theta =\left\{\theta_{1},\ldots ,\theta_{G}\right\}$ resulting from the discretization of the field-of-view, with G≫N: for all arrival angles ${\tilde{\theta }}_{m},m\in \left[1,\ldots ,M\right]$, there exists g∈[1,…,G] such that ${\tilde{\theta }}_{m}=\theta_{g}$. This assumption is often considered in operational systems to measure the calibration table $A=\left[a\left(\theta_{1}\right),\ldots ,a\left(\theta_{G}\right)\right]$ containing the array responses for the angles in Θ. Considering this calibration table, the measurement x(t) can be expressed as:

(5) x(t)=As(t)+n(t),

where $s\left(t\right)\in C^{G}$ is sparse with only *M* non-zero entries corresponding to the sources signals ${\tilde{s}}_{m}\left(t\right)$, with M≪G.

Under the assumption that the sources are uncorrelated, it is interesting to use the vectorized covariance matrix in algorithms: it allows us to consider the contribution of multiple snapshots thus increasing the accuracy, without increasing the computational cost by much. It also has the advantage of being a SMV model, thus all associated methods can be used, and it additionally gives the possibility of estimating more sources than the number of sensors.

### 2.2. Vectorized Covariance Matrix Model

Considering uncorrelated sources, the covariance matrix $R_{xx}\;\hat{=}\;E\left[x\left(t\right)x^{H}\left(t\right)\right]$ is given by:

(6) $$R_{xx}=\sum_{m=1}^{M}a\left({\tilde{\theta }}_{m}\right)a^{H}\left({\tilde{\theta }}_{m}\right){\tilde{\gamma }}_{m}+\sigma_{n}^{2}I_{N},$$

with ${\tilde{\gamma }}_{m}$ the power of the *m*th source and $I_{N}$ the square identity matrix of dimension *N*. The vectorized covariance matrix, noted as $r=\mathrm{vec}(R_{xx})$, is the vector obtained from the concatenation of the columns of $R_{xx}$. It can be expressed as

(7) $$r=\sum_{m=1}^{M}b\left({\tilde{\theta }}_{m}\right){\tilde{\gamma }}_{m}+\sigma_{n}^{2}\mathrm{vec}\left(I_{N}\right)$$

with $b=a^{*}⊗a$, $a^{*}$ is the conjugate of a, and ⊗ is the Kronecker product. Considering a dictionary $B=\left[b\left(\theta_{1}\right),\ldots ,b\left(\theta_{G}\right)\right]$ computed from the calibration matrix A, we have $r=B\gamma +\sigma_{n}^{2}\mathrm{vec}\left(I_{N}\right)$. Considering *K* finite samples, the covariance matrix is estimated by R^xx=1K∑k=1Kx(tk)xH(tk), and we denote $\hat{r}$ the associated estimated vectorized covariance matrix. Let us suppose that the power of the noise is known. We consider the noisy observation vector $y\in C^{N^{2}}$ computed as:

(8) $$y=\hat{r}-\sigma_{n}^{2}\mathrm{vec}\left(I_{N}\right)=B\gamma +w,$$

with $B\in C^{N^{2}\times G}$. The noise vector w results from the estimation of $R_{xx}$ with a finite number of snapshots. The power vector $\gamma \in C^{G}$ is sparse and the indices of non-zero components indicate the directions of the sources. The aim of sparse DOA estimation is to retrieve the indices of non-zero components of vector γ in Equation (8), through the resolution of the problem given by Equation (1).

## 3. Description and Numerical Investigations of the Minimizers of $J_{\ell_{0}}$ and $J_{\mathrm{CEL}0}$

It is known that there are numerous local minima in $J_{\ell_{0}}$, which complicates the minimization of the criterion. Initialization of iterative descent algorithms is particularly delicate, as also highlighted in the DOA estimation literature. In [12], we have successfully used CEL0 penalty for DOA estimation of well-separated sources. Those good results do not seem to be transposed to the case of close sources. It is important now to analyze more deeply the minimizers of $\ell_{0}$ and CEL0 penalized problems in order to propose an optimization scheme able to resolve closer sources.

### 3.1. Simulation Setup

Although our approach is array and scenario independent, we illustrate it in this paper with the following setup. We consider an Uniform Circular Array (UCA) with N=7 antennas and radius d=λ02, where $\lambda_{0}$ is the wavelength. This array could allow for two-dimensions direction-of-arrival estimation, but we limit ourselves to azimuth estimation. The −3 dB beamwidth of this array is 40°. UCAs are well known for their θ invariant performance. We study the case of M=2 incoming sources located at ${\tilde{\theta }}_{1}$ = 32° and a varying ${\tilde{\theta }}_{2}$. The number of snapshots is fixed to K=50. In this part, the received signal is noiseless. The field-of-view is the range [0, 360]° with a grid spacing of 0.5° (G=720). The mutual coherence, corresponding to the maximum absolute correlation between two columns of the dictionary, is in this case close to 1: $\ell_{1}$ methods are thus ineffective.

### 3.2. Minimizers of $J_{\ell_{0}}$

Let us define $I_{G}=\left\{1,\ldots ,G\right\}$ the ordered index set. For a given observation $y\in C^{G}$ and a set $\omega \subseteq I_{G}$, we define the constrained problem $\left(C_{\omega }\right)$ as follows:

(9) $$\left(C_{\omega }\right)\;:\;\min_{\gamma }{∥B\gamma -y∥}_{2}^{2},\;\;s.\;t.\;\;\gamma_{i}=0,\;\forall i\in I_{G}\setminus \omega$$

where $\gamma_{i}$ is the *i*th component of vector γ. Let us note ${\hat{\gamma }}_{\omega }$ the subvector of $\hat{\gamma }$ composed only with the terms indicated by ω: ${\hat{\gamma }}_{\omega }={\left({\hat{\gamma }}_{\omega_{1}},\ldots ,{\hat{\gamma }}_{\omega_{♯\omega }}\right)}^{T}$, with ♯ω the number of elements in ω. The constraint ensures the solutions $\hat{\gamma }$ are sparse vectors whose indices of all non zero entries are in ω, i.e., all components whose indices are not in ω are null. We can then write $\hat{\gamma }=Z_{p}\left({\hat{\gamma }}_{\omega }\right)$, where $Z_{p}$ is a zero padding operator in $I_{G}$:

(10) $${\hat{\gamma }}_{i}=\left\{\begin{array}{cc} 0 & \;\mathrm{if}\;i\notin \omega \\ {\hat{\gamma }}_{\omega_{k}} & \;\mathrm{for}\;\mathrm{the}\;\mathrm{unique}\;k\;\mathrm{such}\;\mathrm{that}\;\omega_{k}=i. \end{array}\right.$$

For any $\omega \subseteq I_{G}$, $\hat{\gamma }\in C^{G}$ solves $\left(C_{\omega }\right)$ if and only if ${\hat{\gamma }}_{\omega }\in C^{♯\omega }$ solves $B_{\omega }^{H}B_{\omega }x=B_{\omega }^{H}y$ and $\hat{\gamma }=Z_{p}\left({\hat{\gamma }}_{\omega }\right)$. $B_{\omega }$ is the submatrix of B composed only with the columns whose indices are in ω.

There are strong connections between the minimizers of the constrained problem $\left(C_{\omega }\right)$ and the minimizers of the regularized criterion $J_{\ell_{0}}\left(\lambda ,\cdot \right)$ that we want to analyze. For $y\in C^{N}$, given a set $\omega \subseteq I_{G}$, let $\hat{\gamma }$ solve problem $\left(C_{\omega }\right)$. Then for any λ, $J_{\ell_{0}}\left(\lambda ,\cdot \right)$ reaches a (local) minimum at $\hat{\gamma }$ (Proposition 2.3 [20]). Conversely, for $y\in C^{N}$ and λ>0, let $J_{\ell_{0}}\left(\lambda ,\cdot \right)$ have a (local) minimum at $\hat{\gamma }$. Then $\hat{\gamma }$ solves $\left(C_{\hat{\omega }}\right)$ for $\hat{\omega }=\mathrm{supp}\left(\hat{\gamma }\right)$ (Lemma 2.4 [20]). Moreover, the (local) minimum that $J_{\ell_{0}}\left(\lambda ,\cdot \right)$ has at $\hat{\gamma }$ is strict iff $\mathrm{rank}\left(B_{\hat{\omega }}\right)=♯\hat{\omega }$ (Theorem 3.2 [20]). There is thus a large number of local minima: the number of supports $\omega \subseteq I_{G}$ such that $\mathrm{rank}(B_{\omega })=♯\omega$ that lead to strict local minima is upper bounded by ∑k=0N2Gk.

In [20], it is shown that under mild conditions, $J_{\ell_{0}}\left(\lambda ,\cdot \right)$ have a unique strict global minimizer. It is common knowledge that the optimal solution of the regularized problem given by Equation (1) depends on the regularization parameter λ, which balances the relative importance between data fidelity and sparsity. In most papers, this parameter is empirically tuned. In previous works [12,13], we proposed a theoretical analysis for an off-line selection of λ. In the sequel of this paper, λ will be selected in an appropriate interval *I* as defined in [12,13].

**Figure 1:**
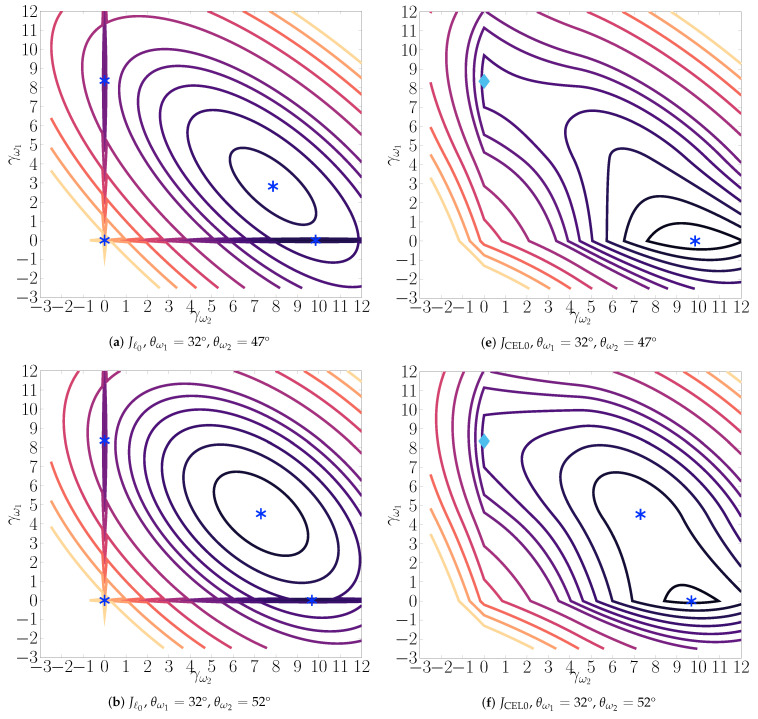

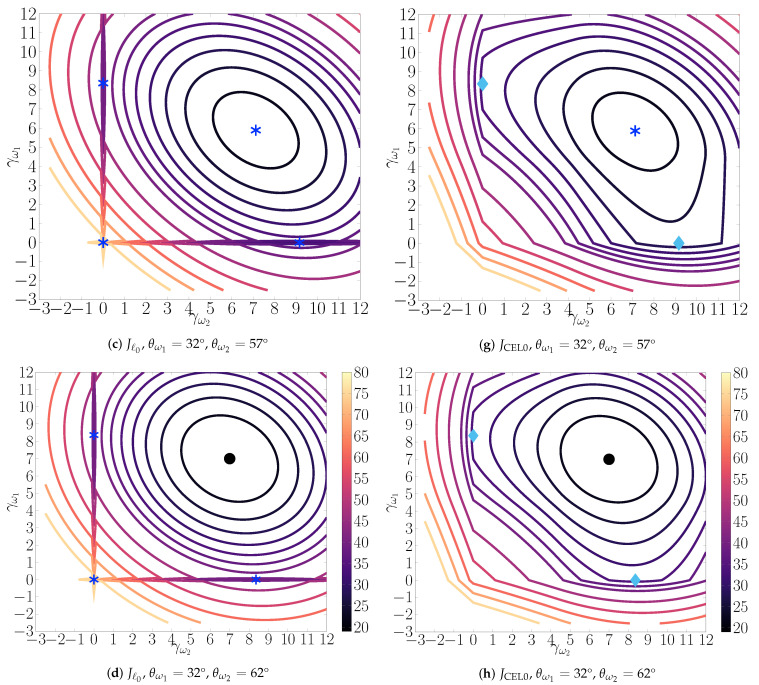
Loss surfaces of $J_{\ell_{0}}$ (**a**–**d**) and $J_{\mathrm{CEL}0}$ (**e**–**h**) as a function of $\gamma_{\omega_{1}}$ and $\gamma_{\omega_{2}}$, for $\gamma =Z_{p}\left(\gamma_{\omega }\right)$, with ♯ω=2, i.e., at most two non-zero components corresponding to directions $\theta_{\omega_{1}}={\tilde{\theta }}_{1}$ and a varying $\theta_{\omega_{2}}$. When those directions correspond to the true ones ${\tilde{\theta }}_{1}$ = 32° and ${\tilde{\theta }}_{2}$ = 62° (**d** and **h**) the global minimum indicated by a black filled circle ($\gamma_{\omega_{1}}=\gamma_{\omega_{2}}=7$) is equal to 2λ=19. Local minima are indicated by the blue asterisks, while light blue diamonds represent critical points that are not local minima.

Figure 1a–d represents projections of $J_{\ell_{0}}$ for the scenario described above, for ${\tilde{\theta }}_{1}$ = 32° and ${\tilde{\theta }}_{2}$ = 62° in the noiseless case. Iso-levels of $J_{\ell_{0}}$ are reported for a vector γ having at most two non-zero components: $\gamma =Z_{p}\left(\gamma_{\omega }\right)$ with ♯ω=2. Those components correspond to fixed directions $\theta_{\omega_{1}}={\tilde{\theta }}_{1}$ = 32° and $\theta_{\omega_{2}}$ which changes on the different figures. On (a), $\theta_{\omega_{2}}$ = 47°, which corresponds to 12θ˜1+θ˜2; on (d), $\theta_{\omega_{2}}={\tilde{\theta }}_{2}$ = 62°. In between, we set: $\theta_{\omega_{2}}$ = 52° and 57°. The values of the two components $\gamma_{\omega_{1}}$ and $\gamma_{\omega_{2}}$, which are the only components allowed to be non-zero, are varying along the two axis. λ is fixed to 9.5, which belongs to the interval *I*. In each figure, we see four (local) minima: the local minimum at 0, local minima along the axis (i.e., one non-zero component), and those corresponding to strictly two non-zero components. The global minimum (black filled circle) is located on Figure 1d for $\gamma_{\omega_{1}}=\gamma_{\omega_{2}}=7$ and its value is 2λ=19.

### 3.3. Minimizers of $J_{\mathrm{CEL}0}$

The global minimizer of $J_{\ell_{0}}$ is preserved in $J_{\mathrm{CEL}0}$, but the number of local minima of $J_{\mathrm{CEL}0}$ may be inferior to the number of local minima of $J_{\ell_{0}}$. Particularly, a local minimum γ of $J_{\mathrm{CEL}0}$ verifies $|\gamma_{i}|\in \left\{0\right\}\cup \left[\sqrt{2\lambda },+\infty \right)$; hence local minimizers of $J_{\ell_{0}}$ having at least one component $|\gamma_{i}|\in \left(0,\sqrt{2\lambda }\right)$ are not local minimizers of $J_{\mathrm{CEL}0}$ [21]. Figure 1e–h represents the loss surfaces of $J_{\mathrm{CEL}0}$. We observe the suppression of local minima on $J_{\mathrm{CEL}0}$ for $\theta_{\omega_{2}}$ close to 12θ˜1+θ˜2 = 47°, for which $0<\gamma_{\omega_{2}}<\sqrt{2\lambda }$ = 4.36. Some local minima of $J_{\ell_{0}}$ are also only critical points of $J_{\mathrm{CEL}0}$. Moreover, the local minima that $J_{\ell_{0}}$ has at 0 is no longer one in $J_{\mathrm{CEL}0}$, which is particularly interesting for the initialization of iterative optimization algorithms.

**Figure 2:**
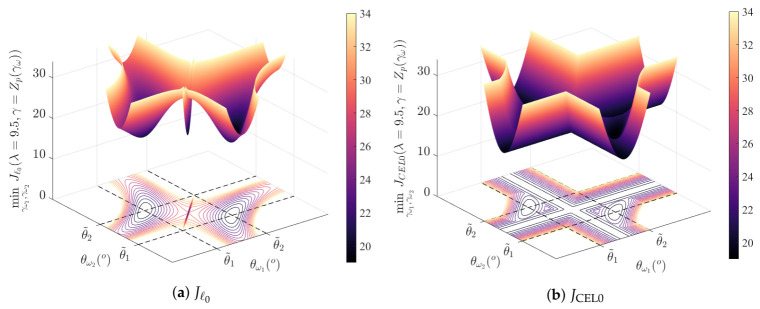
Minimum of the loss surfaces of $J_{\ell_{0}}$ (**a**) and $J_{\mathrm{CEL}0}$ (**b**) for $\gamma =Z_{p}\left(\gamma_{\omega }\right)$, with ♯ω=2, as a function of $\theta_{\omega_{1}}$ and $\theta_{\omega_{2}}$. The diagonal corresponds to $\theta_{\omega_{1}}=\theta_{\omega_{2}}$, i.e., ♯ω=1.

However, those good properties also have a disadvantage: it leads to “flat” minima, i.e., large connected regions where the error remains approximately constant. This is illustrated on Figure 2, comparing the minimum of $J_{\ell_{0}}$ and $J_{\mathrm{CEL}0}$ as a function of $\theta_{\omega_{1}}$ and $\theta_{\omega_{2}}$ for two (or one) non-zero components. Numerous points of the CEL0 surface are approximately at the level of the local minima corresponding to θω1=θω2=12(θ˜1+θ˜2). This behavior appears for close sources when this point is also close to the global minimum.

## 4. Alternating between Loss Surfaces

**Figure 3:**
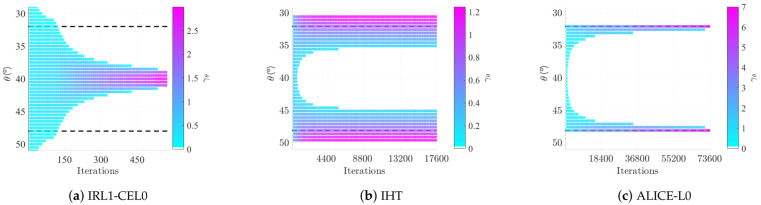
Solutions as iterations go by for close sources with no noise, for λ=0.78. X-axis: iteration number. Y-axis: directions associated with components of $\hat{\gamma }$. The color represents the level of the components. True sources ${\tilde{\theta }}_{1}$ = 32° and ${\tilde{\theta }}_{2}$ = 48° are indicated by the black doted lines. Corresponding components of the optimal solution are equal to 7, all others are null.

For well separated sources, we have shown in [12] than IRL1 algorithm used to minimize $J_{\mathrm{CEL}0}$ (IRL1-CEL0) gives better statistical results than IHT and at a lower computational cost. Indeed, IRL1-CEL0 benefits from the suppression of local minima in this case. Unfortunately, IRL1-CEL0 fails for close sources. This behavior is illustrated on Figure 3a, for true sources at 32° and 48°. Let us note that MUSIC fails for such close sources. The IRL1-CEL0 algorithm is rapidly attracted by a local bad basin corresponding to a few non-zero components for directions in the middle of the true directions (lack of resolution). In this example, denoting $\hat{\gamma }$ the final estimated vector and $\hat{\omega }$ the set indicating the non-zero components, we verify that $B_{\hat{\omega }}^{H}B_{\hat{\omega }}{\hat{\gamma }}_{\hat{\omega }}=B_{\hat{\omega }}^{H}y$ and $\mathrm{rank}\left(B_{\hat{\omega }}\right)=♯\hat{\omega }=5$: it is a strict local minimum of $J_{\ell_{0}}$. The IHT algorithm also remains stuck in a local minimum with this time numerous non-zero components (Figure 3b), forming two clusters around the true directions. In order to avoid being attracted by a bad basin and take advantage of both regularizations, we propose to alternate the minimization between them. Based on this heuristic, we propose the optimization scheme ALICE-L0 (Alternated Landscapes Iterations for Complementary Enhancement for $\ell_{0}$) detailed on Algorithm 1.

**Algorithm 1. Optimization Scheme ALICE-L0** (Alternated Landscapes Iterations for Complementary Enhancement for $\ell_{0}$)**Input:** dictionary B, observation y, β=1L, *L* Lipschitz-constant of B, $\tau_{1}$, $\tau_{2}$, stopping criteria**Initialization:**${\hat{\gamma }}^{\left(0\right)}=0$, i=0, $i_{outer}=0$    • $\epsilon^{\left(i\right)}={∥B{\hat{\gamma }}^{\left(i\right)}-y∥}_{2}^{2}$    **while** $\frac{\left|\epsilon^{\left(i-1\right)}-\epsilon^{\left(i-2\right)}\right|}{\epsilon^{\left(i-1\right)}}>\epsilon_{lim}$ and $i_{outer}<n_{lim}$ **do**        • $i_{outer}=i_{outer}+1$        • Compute w weighting vector by:    $\;\;\;\;w_{g}=(\sqrt{2\lambda }-|\gamma_{g}^{\left(i\right)}|){𝟙}_{\left\{|\gamma_{g}^{\left(i\right)}|\leq \sqrt{2\lambda }\right\}}$, g=1..G        • j=1, $T^{\left(1\right)}=1$, $z^{\left(1\right)}={\hat{\gamma }}^{\left(i\right)}$        **while** $\frac{\left|\epsilon^{\left(i-1\right)}-\epsilon^{\left(i-2\right)}\right|}{\epsilon^{\left(i-1\right)}}>\epsilon_{lim,1}$ and $j<n_{lim,1}$ **do**            (weighted FISTA iterations)            • j=j+1, i=i+1            • ${\hat{\gamma }}^{\left(i\right)}={\mathrm{prox}}_{{∥\cdot ∥}_{1},\lambda \beta w}\left(z^{\left(j-1\right)}-\beta B^{H}\left(Bz^{\left(j-1\right)}-y\right)\right)$            • $T^{\left(j\right)}=\frac{1+\sqrt{1+{\left(2T^{\left(j-1\right)}\right)}^{2}}}{2}$            • $z^{\left(j\right)}={\hat{\gamma }}^{\left(i\right)}+\frac{T^{\left(j-1\right)}-1}{T^{\left(j\right)}}\left({\hat{\gamma }}^{\left(i\right)}-{\hat{\gamma }}^{\left(i-1\right)}\right)$        **end while**        • k=0        **while** $\frac{\left|\epsilon^{\left(i-1\right)}-\epsilon^{\left(i-2\right)}\right|}{\epsilon^{\left(i-1\right)}}>\epsilon_{lim,2}$ and $k<n_{lim,2}$ **do**            (IHT iterations)            • k=k+1, i=i+1            • ${\hat{\gamma }}^{\left(i\right)}={\mathrm{prox}}_{{∥\cdot ∥}_{0},\lambda \beta }\left({\hat{\gamma }}^{\left(i-1\right)}-\beta B^{H}\left(B{\hat{\gamma }}^{\left(i-1\right)}-y\right)\right)$        **end while**        • $\epsilon_{lim,1}=\tau_{1}\epsilon_{lim,1}$, $\epsilon_{lim,2}=\tau_{2}\epsilon_{lim,2}$    **end while**    **return** ${\hat{\gamma }}^{\left(i_{end}\right)}$

We start the minimization considering the CEL0-regularized functional, and using ${\hat{\gamma }}^{\left(0\right)}=0$ as initialization. Indeed, we previously saw that this local minimum in $J_{\ell_{0}}$ is suppressed in $J_{\mathrm{CEL}0}$. Iterations of the weighted Fast Iterative Soft Thresholding Algorithm (weighted FISTA) aim to minimize the convex majorizer of the nonconvex CEL0 functional. For a weighting vector w, iterations use the proximal of the weighted $\ell_{1}$ function, defined component by component as:

(11) $${\left[{\mathrm{prox}}_{{∥\cdot ∥}_{1},\lambda \beta w}\right]}_{g}\left(x\right)=\max\left\{0,1-\frac{\lambda \beta w}{\left|x_{g}\right|}\right\}x_{g}1_{\left\{|x_{g}|\neq 0\right\}}.$$

After some iterations, the estimated vector is used as initialization for IHT, which performs minimization steps over the $\ell_{0}$-regularized cost function. The hard threshold corresponds to the proximal of the $\ell_{0}$-norm, defined by:

(12) $${\left[{\mathrm{prox}}_{{∥\cdot ∥}_{0},\lambda \beta }\right]}_{g}\left(x\right)=x_{g}1_{\left\{|x_{g}|\geq \sqrt{\lambda \beta }\right\}}.$$

We then loop back to alternate between the loss surfaces. The behavior of our algorithm is represented on Figure 3c for close sources (in this article, we set $\epsilon_{lim}=1\times 10^{-6}$, $\epsilon_{lim,1}=1\times 10^{-2}$, $\epsilon_{lim,2}=1\times 10^{-6}$, $n_{lim}=2000$, $n_{lim,1}=200$, $n_{lim,2}=200$, $\tau_{1}=0.9$, $\tau_{2}=1$). In this noiseless case, this is the only algorithm attaining the global minimum of $J_{\ell_{0}}$.

## 5. Statistical Performance

**Figure 4:**
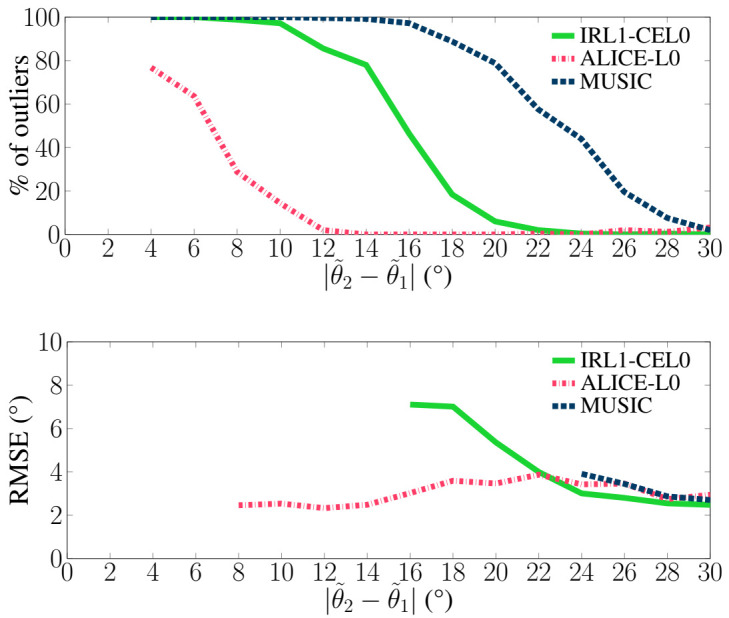
Performance as a function of the sources separation: percentage of outliers and RMSE (not reported when having more than 50% outliers). The regularization parameter λ is fixed to 0.78 according to the theoretical analysis [12].

The purpose of this section is to numerically quantify the algorithms’ performance as a function of the sources’ separation. For that, two criteria will be used: the first one is the percentage of outliers (only one estimated direction or located at more than half a beamwidth of the true directions), and the second one the Root-Mean-Square-Error (RMSE) between estimated and true directions, calculated without outliers. The simulation setup is the one described in Section 3.1, for a Signal-to-Noise Ratio per source equal to 0 dB. Results are presented in Figure 4: we observe that the proposed scheme ALICE-L0 outperforms other methods in terms of statistical accuracy and resolution limit. Let us note that the behavior of IHT is unreliable with noise, and thus statistical results are not presented.

## 6. Conclusions

We linked the operating limits of IHT and IRL1-CEL0 to the properties of corresponding loss landscapes in DOA estimation. To avoid the weaknesses of both criteria, an optimization scheme is proposed using alternatively $J_{\ell_{0}}$ and $J_{\mathrm{CEL}0}$, i.e., alternating between the two regularizations. A particular implementation using λ obtained by [12,13] has been successfully tested, improving for example the resolution limit. Ongoing work concerns algorithm parameters (when to change of regularization) which are here left to the user.

## Data Availability

Data is contained within the article.
